# Efficacy of a Mobile Multidisciplinary Digital Therapeutics App for Patellofemoral Pain: Randomized Controlled Trial

**DOI:** 10.2196/69627

**Published:** 2025-10-17

**Authors:** Sanghee Lee, Chan Yoon, Chi-Hyun Choi, Tae Hyun Park, Sang Jin Yang, Ha Ri Cha, Tae Woo Kim, Jae Hyeon Park, Moon Jong Chang, Chong Bum Chang

**Affiliations:** 1 EverEx Seoul Republic of Korea; 2 Department of Health and Exercise Management University of Tongwon Kwangju Republic of Korea; 3 Department of Orthopedic Surgery Seoul National University College of Medicine SMG-SNU Boramae Medical Center Seoul Republic of Korea; 4 Department of Rehabilitation Medicine Hanyang University College of Medicine Seoul Republic of Korea; 5 Department of Rehabilitation Medicine Hanyang University Guri Hospital Guri-si Republic of Korea; 6 Department of Orthopedic Surgery Seoul National University College of Medicine, Seoul National University Bundang Hospital Gyeonggi-do Republic of Korea

**Keywords:** patellofemoral pain, knee pain, app, smartphone, digital therapeutics, exercise, cognitive behavioral therapy, mobile health, mHealth, eHealth, digital health

## Abstract

**Background:**

Patellofemoral pain (PFP) is a common musculoskeletal disorder characterized by persistent knee pain, often without any structural abnormalities. Conservative treatments, particularly exercise therapy, are widely recommended; however, adherence remains generally low, and full recovery is often not achieved. Psychological interventions can aid in symptom management; however, studies integrating cognitive behavioral therapy (CBT), which is known to be effective for chronic pain, with exercise therapy for patients with PFP are limited. This study examined the impact of MORA Cure (PFP), a multidisciplinary digital therapeutics (DTx) app that integrates exercise and CBT, in comparison with conventional treatments for PFP management.

**Objective:**

This study aimed to evaluate the efficacy and safety of an 8-week DTx intervention incorporating exercise and CBT compared with in-person exercise education in patients with PFP.

**Methods:**

A parallel-group randomized controlled trial was conducted with 35 patients diagnosed with PFP recruited from orthopedic outpatient clinics. Participants were randomly assigned to either the DTx group (n=18, 51%) or the control group (n=17, 49%). The DTx group received an 8-week intervention via the MORA Cure (PFP) app incorporating home-based exercises and weekly CBT modules with daily worksheets. The control group received conventional treatment, including disease education, a single in-person exercise education session conducted by a medical professional, and encouragement to continue self-exercising throughout the study period. The outcome measures included pain severity (usual and worst, assessed using the numeric pain rating scale), functional disability (Anterior Knee Pain Scale), knee extension strength (measured using an isokinetic dynamometer), health-related quality of life (EQ-5D), and mental health status (9-item Patient Health Questionnaire). Assessments were conducted from baseline at 4-week intervals for up to 12 weeks.

**Results:**

The DTx group showed significant reductions in usual pain at each time point (4 weeks: mean score 2.2, SD 1.5, and *P*=.006; 8 weeks: mean 2.3, SD 1.7, and *P*=.003; 12 weeks: mean 1.2, SD 1.8, and *P*=.008), whereas the control group exhibited no changes. The knee extension strength in the DTx group increased significantly at both 8 and 12 weeks (*P*<.001), with greater improvement than that in the control group at 8 weeks (*P*=.04). Both groups showed significant improvements in functional disability at 12 weeks (DTx: mean score 85.2, SD 12.7, and *P*=.006; control: mean 84.5, SD 13.0, and *P*=.01). Health-related quality of life (EQ-5D) also improved in the DTx group at 8 and 12 weeks, whereas the control group showed improvement only at 12 weeks.

**Conclusions:**

This multidisciplinary DTx intervention was associated with significant pain reduction, improved functional disability, and increased knee extension strength in patients with PFP. These findings underscore the promise of DTx in PFP management and their potential to enhance patient outcomes.

**Trial Registration:**

ClinicalTrials.gov NCT05614583; https://clinicaltrials.gov/study/NCT05614583

## Introduction

### Background

Patellofemoral pain (PFP) is one of the most common musculoskeletal disorders causing knee pain, with an annual prevalence of 23% in the general population [1,2]. Although patients with PFP do not exhibit significant structural abnormalities, symptoms often persist for more than 2 years without spontaneous resolution [3,4]. Furthermore, PFP may contribute to the development of patellofemoral osteoarthritis [5-7]. Therefore, active management of PFP is imperative.

Conservative interventions are generally recommended for managing PFP, with therapeutic exercise targeting the hip and lower-extremity muscles being considered the primary intervention [1,7,8]. Despite these recommendations, a considerable proportion of patients in clinical practice exhibit poor adherence to exercise therapy and often fail to achieve complete perceived recovery even after undergoing exercise [9-11]. In addition, some studies have explored the efficacy of psychological interventions in managing PFP because of the association between psychological factors and pain [12-14]. However, few studies have examined the use of cognitive behavioral therapy (CBT), which has demonstrated beneficial effects on chronic pain [15], in combination with exercise therapy for patients with PFP. Therefore, research into interventions that can enhance the effectiveness of exercise therapy and incorporate CBT is required.

In recent years, digital therapeutics (DTx) have emerged as promising treatment options for managing various musculoskeletal conditions [16-20]. A previous meta-analysis demonstrated that DTx enhanced adherence to therapeutic exercises in patients with musculoskeletal disorders [21]. Furthermore, DTx can offer not only therapeutic exercise but also a variety of treatments, including exercise, education, and CBT. Multidisciplinary DTx have been shown to be effective in treating chronic low back and shoulder pain [18,20,22].

### Objectives

However, to our knowledge, research on the effectiveness of multidisciplinary DTx interventions in patients with PFP is lacking. Therefore, this study aimed to investigate the efficacy of an 8-week multidisciplinary mobile app that provides therapeutic exercise, education, and CBT compared to in-person detailed exercise education in patients with PFP in a randomized controlled trial (RCT). We hypothesized that this digital intervention, compared to conventional in-person exercise education, would lead to symptom improvement in patients with PFP after a 12-week follow-up period.

## Methods

### Study Design and Randomization

This study was designed as a parallel-group, individual RCT to evaluate the safety and efficacy of a multidisciplinary DTx app, MORA Cure (PFP), compared with conventional treatment for PFP. The trial was conducted at the Department of Orthopedics, Seoul National University Bundang Hospital, and Boramae Medical Center. The study protocol, along with the statistical analysis plan, has been previously published and registered at ClinicalTrials.gov (NCT05614583).

The participants were recruited from outpatient clinics between November 2022 and July 2023. Study-related posters were displayed in clinic waiting areas, and individuals who responded to the information provided in the posters were screened by the research team for eligibility. Those who met the inclusion criteria and provided written informed consent were enrolled in the study. Participants were randomly assigned in a 1:1 ratio to either the DTx group, which received the MORA Cure (PFP) DTx intervention, or the control group, which received conventional treatment. Randomization was performed using a computer-generated block randomization method. An independent statistician generated the randomization sequence, and allocation concealment was ensured using sealed, opaque envelopes. To minimize bias, the outcome assessors were blinded to group allocation.

### Study Participants

The inclusion criteria for this study were as follows: adults aged 19 to 49 years with a history of PFP lasting for at least 3 months but less than 2 years affecting at least one knee joint. The diagnosis of PFP was made by orthopedic surgeons based on clinical symptoms, including anterior or retropatellar knee pain, in conjunction with physical examination findings and plain radiographic imaging to exclude structural abnormalities. Standardized anteroposterior weight-bearing knee radiographs with approximately 10° of flexion were obtained to rule out conditions such as osteoarthritis or bony lesions. If both knees were affected, the knee with the highest pain score on the numeric pain rating scale (NPRS) was selected as the target joint for evaluation; this selection was maintained throughout the trial. The participants were also required to report pain during squatting and at least 2 of the following activities: prolonged sitting, cycling, running, stair climbing, kneeling, patellar compression, and palpation of the patellar facets.

The exclusion criteria were knee osteoarthritis classified as Kellgren-Lawrence grade 2 or higher on radiographs, history of significant knee injuries (including fractures, ligament injuries, cartilage damage, or patellar dislocation), or previous knee surgery. Participants diagnosed with patellar tendinitis or structural knee abnormalities (such as Osgood-Schlatter disease), who had conditions that could limit physical activity, who were pregnant or breastfeeding, who used pain or anti-inflammatory medications for other conditions, and who had a history of substance or alcohol abuse were also excluded. Participants with psychiatric conditions that could affect pain perception, such as somatoform disorders, were excluded.

During the trial, intra-articular injections, surgery, narcotic analgesics, topical agents, and any treatment intended to alleviate joint pain outside the trial protocol were prohibited. However, acetaminophen (650 mg) was allowed as a rescue medication at a dosage of 2 tablets every 8 hours (up to a maximum of 6 tablets per day). The participants were instructed to avoid acetaminophen within 24 hours before each visit and until all assessments were completed.

### Ethical Considerations

This study was approved by the institutional review boards of Seoul National University Bundang Hospital (E-2209-780) and Boramae Medical Center (10-2023-33). This study was conducted in accordance with the ethical principles outlined in the Declaration of Helsinki. Written informed consent was obtained from all participants before enrollment, and the investigators ensured adherence to the trial procedures and protocol. Participants were informed of their right to voluntarily participate and withdraw from the study at any time without penalty. All data were deidentified before analysis and stored in password-protected files accessible only to the research team in accordance with institutional data protection policies. No personally identifiable information was used in the analysis or dissemination of results. Participants received a transportation allowance of 50,000 KRW (approximately US $36) at each visit. No additional financial compensation was offered for participation.

### Intervention and Control Groups

**Figure 1:**
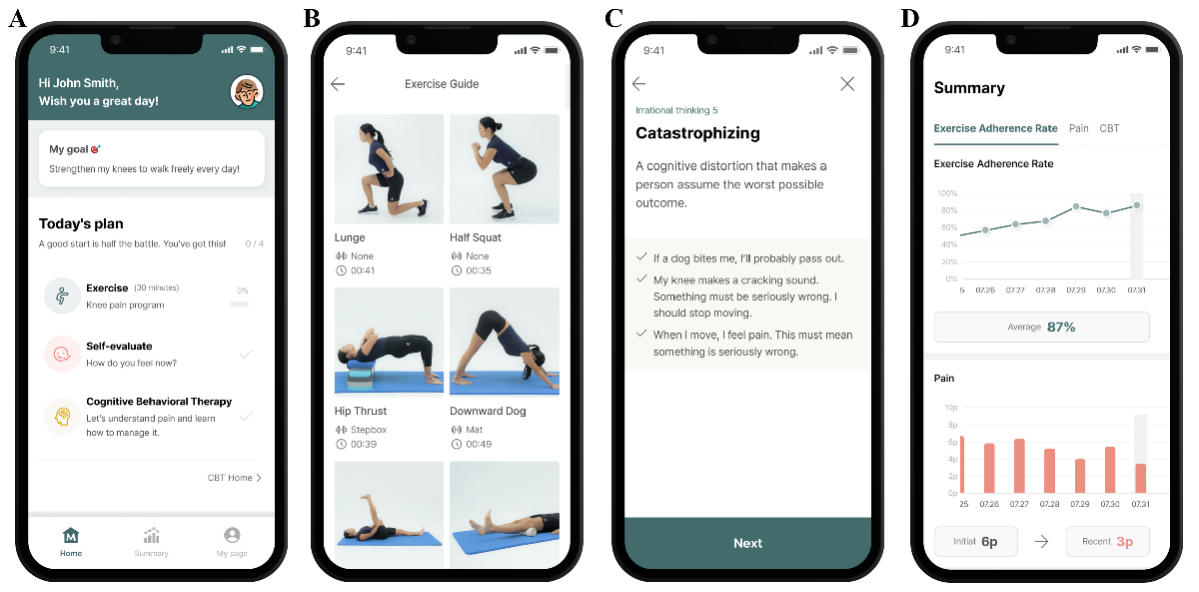
Multidisciplinary digital therapeutics MORA Cure (PFP), incorporating personalized exercise therapy and self-guided cognitive behavioral therapy for patellofemoral pain.

The DTx group used the MORA Cure (PFP) DTx app for 8 weeks. Participants were provided with free access to the app as part of the study and were guided by the research team during enrollment. Figure 1 shows a schematic of the MORA Cure (PFP) multidisciplinary DTx model. The intervention included a structured home-based exercise program incorporating combined hip- and knee-targeted muscle strengthening and stretching exercises as recommended by the clinical guidelines for PFP [23,24]. The exercises were designed to progressively strengthen the knee and hip muscles, with intensity adjusted according to the participants’ reported pain levels as assessed using the Borg rating of perceived exertion scale and the NPRS. Participants completed daily exercise sessions lasting approximately 30 minutes.

In addition to exercise therapy, the participants received weekly CBT modules. These modules covered topics such as identifying and managing negative emotions and pain-related thoughts, developing positive coping strategies, and practicing relaxation techniques. Throughout with the 8 weekly modules, participants were required to complete daily worksheets to reinforce their understanding and practice of the CBT content.

Following the 8-week treatment period, the exercise therapy materials remained accessible via the app for continued use. The participants were encouraged to continue their exercise routine throughout the observation period.

The control group received conventional treatment, including disease education materials and a 30-minute, in-person exercise education session with a medical professional specializing in musculoskeletal disorders. The participants were provided with educational materials outlining the instructed content and were encouraged to perform rehabilitation exercises independently at home.

### Outcome Measures

The primary outcomes of this study were changes in the usual pain severity and functional disability. Usual pain severity was assessed using the NPRS, in which the participants rated their average daily pain over the previous week on a scale from 0 (no pain) to 10 (worst pain imaginable). The second primary outcome, functional disability, was measured using the Anterior Knee Pain Scale (AKPS), also known as the Kujala score. This disease-specific disability scale ranges from 0 (severe disability) to 100 (no disability) and assesses the limitations specific to PFP. Outcome measures were collected through in-person evaluations conducted at the clinical sites.

Secondary outcomes included assessments of the worst pain experienced in the previous week and pain during specific activities, such as running, sit-to-stand transitions, stair ascent, and stair descent. Participants’ health-related quality of life (QoL) was measured using the EQ-5D. Psychological outcomes were assessed using the 9-item Patient Health Questionnaire (PHQ-9) to evaluate depressive symptoms. The knee extensor strength was measured using a Biodex isokinetic dynamometer (Biodex Medical Systems, Inc). Usual pain, worst pain, functional disability, and health-related QoL were assessed at baseline and at 4, 8, and 12 weeks. Knee extensor strength and psychological outcomes were assessed at baseline and 8 and 12 weeks.

In addition to the primary and secondary outcomes, baseline demographic and structural alignment variables, including age, sex, BMI, pain duration, and the hip-knee-ankle (HKA) angle, were collected at enrollment. The HKA angle, defined as the angle formed by lines connecting the centers of the femoral head, knee joint, and talus, was measured using standardized weight-bearing full-length anteroposterior radiographs [25]. Malalignment in the coronal plane, as quantified by the HKA angle, has been associated with patellofemoral joint degeneration and instability and may play a role as a biomechanical contributor to PFP [26-28].

### Compliance and Safety Monitoring

Treatment compliance was monitored and compared between the DTx and control groups. In the DTx group, treatment compliance was evaluated at weeks 4 and 8 based on the actual time participants spent exercising, as recorded by the app, relative to the prescribed target time. In the control group, the participants documented their daily exercise completion and session duration in a log, which was reviewed at each visit to monitor adherence throughout the study. Participants were advised to complete 30 minutes of exercise 6 times per week. Compliance in the control group was evaluated based on the total weekly exercise time relative to the recommended exercise duration. Safety was monitored throughout the study by recording adverse events or symptom exacerbations reported by participants. At each visit (weeks 4, 8, and 12), participants were asked about any new or worsening symptoms related to the intervention and were encouraged to report adverse events at any time during the trial.

### Sample Size

Estimating the target sample size for this trial was challenging due to the lack of previous studies comparing the efficacy of DTx integrating both exercise and CBT for PFP. To guide the calculation, we referred to a previous prospective study that evaluated the effectiveness of in-person therapy combining exercise therapy with psychological intervention in a similar patient population [13]. On the basis of this study’s results, where the primary outcome of usual pain showed a significant difference between groups (mean difference −6.5, 95% CI −10.9 to −2.1; *P*=.001; effect size=1.11), we calculated the sample size.

Sample size calculation was conducted using the G*Power software with the following parameters: type I error (α) of .05, 80% power, and the effect size derived from a previous study. This calculation yielded the required sample size of 14 participants per group. Considering previous studies with an 8-week intervention period and accounting for a potential dropout rate of up to 30%, we aimed to recruit a total of 40 participants, with 20 in each group [29].

### Statistical Analysis

The primary analysis was conducted on the per-protocol population, which comprised 13 participants per group (DTx and control). Statistical analyses were conducted using the R software (version 4.4.1; R Foundation for Statistical Computing).

Baseline characteristics were compared between the groups using appropriate statistical tests to assess differences. For continuous variables, either the 2-tailed *t* test or Mann-Whitney *U* test was used depending on the normality of the data distribution. As sex was the only categorical variable, the Fisher exact test was used to assess between-group differences. Between-group comparisons of change scores (eg, baseline to 8 weeks) were conducted using the Mann-Whitney *U* test.

Within-group differences from baseline to the postintervention time point were assessed using the Friedman test for repeated measures. Post hoc pairwise comparisons were conducted using the Wilcoxon signed rank test, with the Bonferroni correction applied to adjust the significance threshold for multiple comparisons.

A *P* value of <.05 was considered statistically significant for all analyses. When the Bonferroni correction was applied to account for multiple comparisons, the significance threshold was adjusted by dividing the conventional α level (.05) by the number of comparisons, controlling for familywise errors, and reducing the risk of type I errors.

## Results

### Study Population

**Figure 2:**
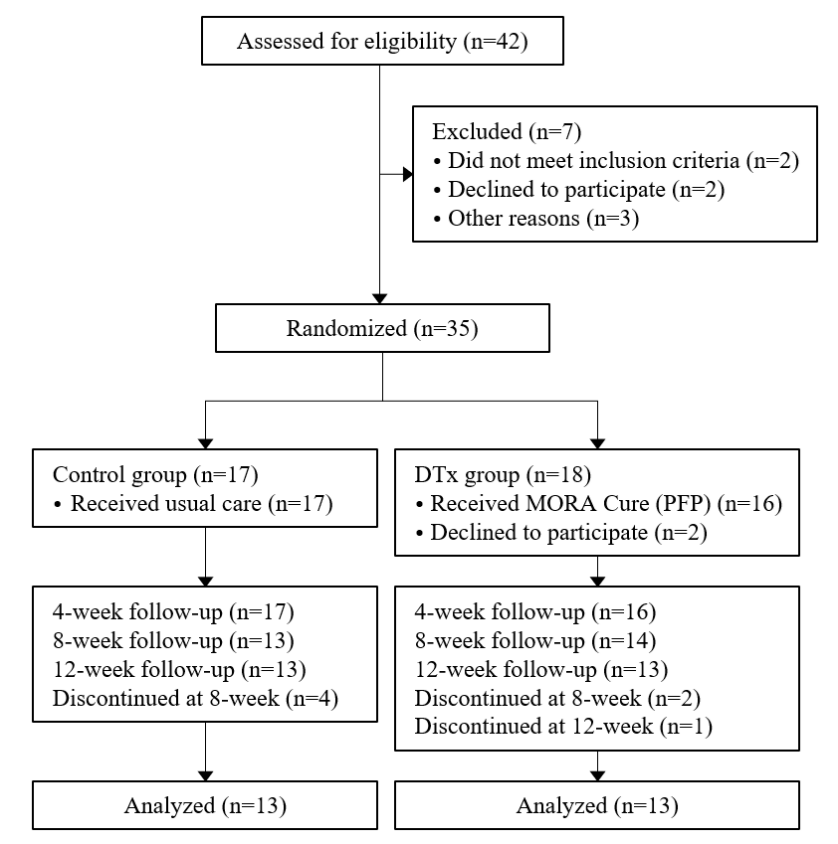
CONSORT (Consolidated Standards of Reporting Trials) diagram. DTx: digital therapeutics.

Of the 42 individuals screened for eligibility, 7 (17%) were excluded: 2 (29%) for not meeting the inclusion criteria (one due to PFP duration and the other due to knee osteoarthritis); 2 (29%) for declining participation; and 3 (43%) for other reasons, including vulnerability and follow-up issues (Figure 2). This resulted in 35 participants randomized into the control (n=17, 49%) and DTx (n=18, 51%) groups. In the DTx group, of the 18 participants, 16 (89%) began the intervention, with 2 (11%) declining to participate after randomization. By the 12-week follow-up, 72% (13/18) of the participants remained in the DTx group for analysis after 3 withdrawals. In the control group, all 17 participants remained up to the 4-week follow-up; however, 4 (24%) discontinued their participation by the 8-week follow-up due to consent withdrawal and worsening knee pain, resulting in 13 (76%) participants for the final analysis. Ultimately, 13 participants in each group completed all the assessments over the 12-week period.

A total of 26 participants (n=13, 50% per group) were included in the final analysis. The baseline characteristics of the 2 groups were largely comparable across key clinical variables, including age, sex, BMI, HKA angle, pain scores, and knee function, except for PHQ-9 scores (Table 1). PHQ-9 scores, which assess depressive symptoms, were significantly higher in the control group than in the DTx group (*P*=.02). Participants were mostly women in their mid-30s, and the average duration of knee pain was approximately 1 year (DTx group: mean 48.4, SD 22.0 weeks; control group: mean 49.7, SD 26.3 weeks). The mean usual pain score at baseline was 3.9 (SD 2.0) in the DTx group and 3.7 (SD 1.5) in the control group, whereas the mean worst pain score was 4.8 (SD 2.5) and 5.2 (SD 1.9), respectively.

**Table 1:** Baseline characteristics and symptom-related variables of the study participants^a^.

Variable	DTx^b^ group (n=13)	Control group (n=13)	*P* value
Age (y), mean (SD)	33.6 (11.0)	34.8 (10.9)	.76
Sex (female), n (%)	11 (84.6)	9 (69.2)	.65
BMI (kg/m^2^), mean (SD)	21.3 (1.9)	21.3 (2.0)	.80
HKA^c^ angle (°), mean (SD)	−0.6 (3.4)	−0.7 (3.8)	.92
Usual pain score (0-10), mean (SD)	3.9 (2.0)	3.7 (1.5)	.75
Worst pain score (0-10), mean (SD)	4.8 (2.5)	5.2 (1.9)	.98
Duration of pain (wk), mean (SD)	48.4 (22.0)	49.7 (26.3)	.84
AKPS^d^ score (0-100), mean (SD)	74.5 (11.8)	73.5 (12.8)	.84
Knee extension peak torque (Biodex isokinetic dynamometer; Nm), mean (SD)	78.0 (19.4)	68.5 (32.2)	.09
EQ-5D score (–0.07 to –1.00), mean (SD)	0.8 (0.05)	0.8 (0.05)	.37
PHQ-9^e^ score (0-27), mean (SD)	1.5 (2.2)	3.8 (3.4)	.02

^a^*P* values for continuous variables, including age, BMI, worst pain score, pain duration, knee extension peak torque (measured using a Biodex isokinetic dynamometer), EQ-5D score, and PHQ-9 score, were calculated using the Mann-Whitney *U* test, whereas *P* values for HKA angle, usual pain score, and AKPS score were calculated using the Student *t* test. The *P* value for the sex variable was calculated using the Fisher exact test.

^b^DTx: digital therapeutics.

^c^HKA: hip-knee-ankle.

^d^AKPS: Anterior Knee Pain Scale.

^e^PHQ-9: 9-item Patient Health Questionnaire.

### Outcome Measures

**Figure 3:**
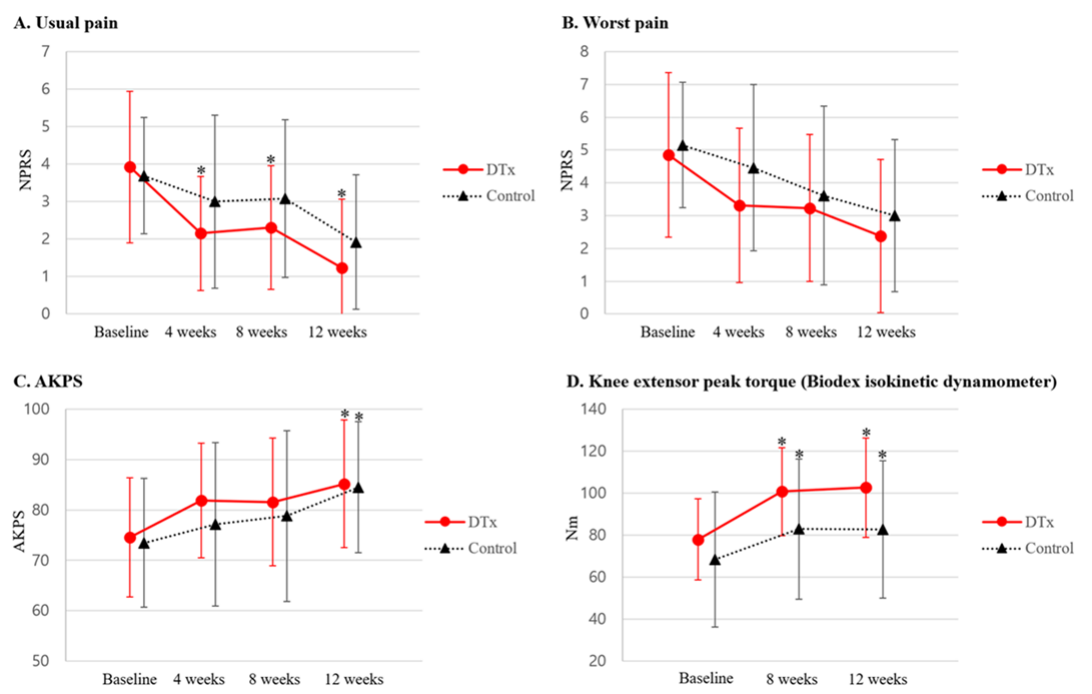
Comparison of clinical outcomes between the digital therapeutics (DTx) and control groups over 8 weeks of treatment. AKPS: Anterior Knee Pain Scale; NPRS: numerical pain rating scale.

Figure 3 illustrates the changes in usual and worst pain, functional disability, and knee extension strength. After applying Bonferroni correction, statistical significance was set at *P*<.02 for usual pain, worst pain, and AKPS and at *P*<.03 for knee extension peak torque. The DTx group showed a statistically significant reduction in usual pain from baseline at all time points. Specifically, usual pain decreased significantly at 4 (mean 2.2, SD 1.5; *P*=.006), 8 (mean 2.3, SD 1.7; *P*=.003), and 12 (mean 1.2, SD 1.8; *P*=.008) weeks in the DTx group. In contrast, the control group did not show significant reductions in pain at 4 (mean 3.0, SD 2.3; *P*=.15), 8 (mean 3.1, SD 2.1; *P*=.32), or 12 (mean 1.9, SD 1.8; *P*=.03) weeks.

For the worst pain, both groups showed a gradual reduction over time; however, after Bonferroni correction, this reduction was not statistically significant from baseline, although the DTx group exhibited borderline significance. In the DTx group, pain reduction was observed at 4 (mean 3.3, SD 2.4; *P*=.03), 8 (mean 3.2, SD 2.2; *P*=.03), and 12 (mean 2.4, SD 2.3; *P*=.04) weeks. The control group showed reductions at 4 (mean 4.5, SD 2.5; *P*=.27), 8 (mean 3.6, SD 2.7; *P*=.12), and 12 (mean 3.0, SD 2.3; *P*=.049) weeks; however, these were not statistically significant.

AKPS scores showed gradual improvement in both groups over time, with significant improvements from baseline observed only at 12 weeks in the DTx (mean 85.2, SD 12.7; *P*=.006) and control (mean 84.5, SD 13.0; *P*=.01) groups. For pain and knee function, between-group comparisons of change scores from baseline showed no statistically significant differences in usual pain (*P*=.19, .69, and .36), worst pain (*P*=.55, .80, and .78), or AKPS scores (*P*=.36, .92, and .72) at 4, 8, and 12 weeks, respectively.

In the DTx group, knee extension peak torque increased significantly from baseline at both 8 (mean 100.8, SD 20.9; *P*<.001) and 12 (mean 102.7, SD 23.7; *P*<.001) weeks. The control group also exhibited significant improvements at 8 (mean 82.9, SD 33.5; *P*=.01) and 12 (mean 82.8, SD 32.7; *P*=.02) weeks. Between-group comparisons in change from baseline revealed that the DTx group showed significantly greater improvements at 8 weeks (*P*=.04), with borderline significance at 12 weeks (*P*=.05).

Table 2 summarizes the changes in pain scores during stair activities, sit-to-stand transitions, and running, as well as the EQ-5D and PHQ-9 scores over time for each group. In the DTx group, pain during stair ascent decreased significantly at both 4 and 12 weeks compared to baseline, whereas stair descent pain showed consistent reductions at all time points. Pain during running was significantly lower at 8 weeks in the DTx group, whereas the control group showed a significant reduction at both 4 and 12 weeks. Regarding health-related QoL, the EQ-5D scores in the DTx group improved significantly at 8 and 12 weeks, whereas the control group showed a similar significant improvement only at 12 weeks. The PHQ-9 scores were significantly reduced in the control group at 12 weeks, with no corresponding changes in the DTx group.

**Table 2:** Comparison of baseline and posttreatment outcomes in the digital therapeutics (DTx) and control groups at multiple time points^a^.

Variable and group			Baseline, mean (SD)		4 wk, mean (SD)		8 wk, mean (SD)		12 wk, mean (SD)
**Pain during stair ascent** **(0-10)**									
	DTx	3.7 (2.4)		2.3 (2.0)		2.2 (2.0)		1.3 (1.6)	
	*P* value	—^b^		.01		.03		.008	
	Control	3.4 (2.1)		2.5 (2.1)		2.2 (1.8)		1.4 (1.4)	
	*P* value	—		.19		.12		.02	
**Pain during stair descent** **(0-10)**									
	DTx	4.0 (2.2)		2.5 (2.1)		2.1 (1.6)		1.7 (1.5)	
	*P* value	—		.007		.006		.008	
	Control	4.0 (2.1)		3.2 (2.3)		2.8 (2.4)		2.2 (2.4)	
	*P* value	—		.17		.09		.03	
**Pain during sit-to-stand transition** **(0-10)**									
	DTx	2.8 (2.6)		1.9 (1.9)		1.3 (1.4)		0.8 (1.3)	
	*P* value	—		.13		.03		.02	
	Control	3.7 (2.3)		2.2 (2.3)		2.4 (1.9)		1.4 (1.8)	
	*P* value	—		.07		.09		.02	
**Pain during running** **(0-10)**									
	DTx	4.5 (2.7)		2.9 (2.2)		2.5 (1.7)		2.2 (2.0)	
	*P* value	—		.03		.009		.03	
	Control	4.7 (2.0)		3.5 (2.0)		3.1 (2.1)		2.5 (2.1)	
	*P* value	—		.01		.03		.01	
**EQ-5D** **(–0.07 to 1.00)**									
	DTx	0.84 (0.05)		0.86 (0.04)		0.87 (0.04)		0.88 (0.04)	
	*P* value	—		.04		.01		.01	
	Control	0.82 (0.05)		0.85 (0.05)		0.85 (0.04)		0.88 (0.04)	
	*P* value	—		.02		.19		.009	
**PHQ-9^c^ (0-27)**									
	DTx	1.5 (2.2)		—		1.7 (2.4)		1.5 (1.6)	
	*P* value	—		—		>.99		.80	
	Control	3.8 (3.4)		—		2.5 (3.2)		1.4 (1.4)	
	*P* value	—		—		.07		.003	

^a^Wilcoxon signed rank tests with Bonferroni corrections were applied for post hoc pairwise comparisons. The adjusted significance level was set at *P*<.02 (.05/3) except in the case of the PHQ-9, where the significance level was *P*<.03 (.05/2).

^b^Baseline values served as the reference for within-group comparisons; therefore, no *P* values were calculated for baseline columns. The PHQ-9 was measured only at baseline, 8 weeks, and 12 weeks; thus, data were not available for the 4-week time point.

^c^PHQ-9: 9-item Patient Health Questionnaire.

### Compliance and Safety Monitoring

At the 4-week assessment, average treatment compliance was 81.4% (SD 24.1%) and 83.7% (SD 74.8%) in the DTx and control groups, respectively (*P*=.84); at 8 weeks, average compliance was 61.9% (SD 29.9%) and 58.4% (SD 42%) in the DTx and control groups, respectively (*P*=.81). No significant differences were observed in compliance between groups at either time point.

No serious adverse events were observed. Mild musculoskeletal symptoms, such as arthralgia, extremity pain, joint stiffness, and muscle spasms, were observed as adverse device effects, with no statistically significant differences between the groups. These symptoms occurred in 15% (2/13) of the participants in the DTx group and in none in the control group, and all events were resolved without complications.

## Discussion

### Principal Findings

The effectiveness of an 8-week multidisciplinary DTx intervention was evaluated in patients with PFP and compared with usual care. This study demonstrated that the DTx app improved pain, functional disability, QoL, and knee extensor strength. Although functional disability, QoL, and knee extensor strength also improved in the control group, significant pain reduction was not observed. However, no significant differences were observed between the 2 groups except for knee extensor strength at week 8.

The results of this study were consistent with those of previous studies demonstrating the efficacy of digital health interventions on musculoskeletal disorders, with a positive impact observed following their application [30,31]. Among these, home-based physical exercise programs were the most commonly used interventions, followed by education, CBT, and monitoring of outcomes or physical activity [30]. A study that implemented a multidisciplinary DTx intervention with the same components (combining therapeutic exercise and CBT) as those used in this study for patients with chronic low back pain also observed reductions in pain and functional disability, although the differences were not statistically significant compared to the control group [32]. However, in previous studies, digital interventions for the knee have primarily been applied to knee osteoarthritis or postarthroplasty rehabilitation, whereas the application of multidisciplinary DTx interventions to PFP remains limited.

For PFP, exercise is the key component of treatment [8,33,34]. Although the optimal exercise for PFP has not yet been determined, combining hip and knee exercises has been reported to be more effective than knee exercises alone [34,35]. A previous meta-analysis reported pain reduction with a mean difference of −1.71 on a 10-point visual analogue scale in patients with PFP following hip and knee exercise programs [35]. In this study, the mean difference in usual pain improved significantly by −1.6 and −2.7 after 8 and 12 weeks, respectively, in the DTx group. The hip and knee exercise studies included in the aforementioned meta-analysis implemented supervised exercise [35]. Therefore, the effect of DTx on reducing usual pain in this study appears comparable to that of supervised exercise. The proposed physiological mechanisms of exercise include reducing pressure on the knee through improved leg strength, promoting tissue adaptation through appropriate loading, and enhancing movement control [24,36]. Although there was no significant difference between the groups, the control group did not show a significant improvement in usual pain. The control group also received 30 minutes of in-person exercise education, and their compliance was adequate. This finding is consistent with those of previous studies that demonstrated that supervised progressive exercise was more effective [37,38]. Adjusting exercise intensity and type using DTx rather than simply performing the same prescribed exercises may have contributed to better pain outcomes.

Patients with PFP often exhibit impaired physical function [39]. Exercise has positive effects on physical disability and strength [34,35]. Although functional disability and pain intensity have been reported to have a moderate relationship [40], both groups showed significant improvement in functional disability by week 12, with no significant difference between the groups. However, the control group did not experience a significant pain reduction. These findings are consistent with those of a previous study that showed that, although there was a significant long-term difference in pain improvement between the control group (who only performed isometric quadriceps contractions and received education) and supervised exercise therapy group, long-term differences in functional disability were not observed [9]. In addition, in terms of knee extensor strength, which is an objectively measured physical function, the DTx group in our study demonstrated significantly greater improvement than the control group by week 8.

Mental distress, including depression, anxiety, and pain catastrophizing, is prevalent among patients with PFP and is often associated with increased pain, impaired physical function, and poor outcomes [12,41,42]. In addition, a previous meta-analysis reported that patients with PFP have a reduced QoL [43]. However, the efficacy of psychological interventions in patients with PFP remains inconclusive [13,14,29]. CBT, one of the most studied psychological approaches in chronic pain management, has shown efficacy in altering maladaptive pain-related thoughts and reducing pain-related avoidant behaviors [15]. These effects are thought to occur via mechanisms such as reduced pain catastrophizing and increased pain coping strategies. In this study, despite randomization, baseline PHQ-9 scores differed between the groups; however, most participants were clinically categorized as having minimal or no depression (score of 0-4; DTx group: 12/13, 92%; control group: 9/13, 69%) [44]. Furthermore, improvement in PHQ-9 scores was observed only in the control group, which may be attributed to baseline differences. Given the potential benefits of psychological interventions, future studies are required to evaluate the effectiveness of DTx for patients with musculoskeletal disorders, particularly for patients with PFP experiencing mental distress.

Given the advances in digital and communication technologies, DTx have been investigated for various musculoskeletal disorders [18,20,45,46]. DTx could enhance overall health outcomes, accessibility, adherence, and cost-effectiveness [21,47,48]. Previous studies have evaluated the effectiveness of digital health technology in knee osteoarthritis and other nonoperative knee conditions and reported positive effects on pain, physical function, and QoL [46,49]. However, RCTs investigating the effects of multidisciplinary DTx, specifically in patients with PFP, are still lacking. Considering the disease characteristics of PFP and our results, multidisciplinary DTx appear promising for the treatment of PFP.

### Limitations

This study has several limitations. First, although the sample size calculation accounted for a 30% dropout rate, the final per-protocol analysis included only 13 participants per group (reflecting a 5/40, 13% prerandomization dropout rate and a 9/35, 26% postrandomization dropout rate) due to additional attrition, which may have reduced the statistical power and limited the generalizability of the findings. Second, participant blinding was not feasible due to the nature of the intervention. Third, many participants reported low baseline pain and PHQ-9 scores, which could have influenced their responsiveness to treatment. Fourth, despite randomization, there was a baseline imbalance in PHQ-9 scores between groups, potentially confounding the psychological outcomes. Fifth, hip strength, which plays an important role in PFP management, was not assessed. Sixth, because the intervention combined exercise and CBT, it is difficult to determine the relative contribution of each component to the observed effects. Finally, the 12-week follow-up period may have been insufficient to evaluate the long-term sustainability of treatment outcomes.

### Conclusions

This study demonstrated that an 8-week multidisciplinary DTx intervention incorporating exercise and CBT effectively reduced pain, enhanced knee extensor strength, and improved QoL in patients with PFP. These findings support the efficacy and safety of a multidisciplinary DTx approach for PFP, highlighting its potential to enhance patient adherence and facilitate symptom management through accessible home-based interventions. Further large-scale studies are required to explore the broad applicability and sustained effectiveness of DTx for PFP.

## Data Availability

The datasets generated or analyzed during this study are available from the corresponding author on reasonable request.

## Appendix

Multimedia Appendix 1 CONSORT-eHEALTH checklist (V 1.6.1).

## Abbreviations

AKPS: Anterior Knee Pain Scale
CBT: cognitive behavioral therapy
DTx: digital therapeutics
HKA: hip-knee-ankle
NPRS: numeric pain rating scale
PFP: patellofemoral pain
PHQ-9: 9-item Patient Health Questionnaire
QoL: quality of life
RCT: randomized controlled trial
